# Management of toxicities from antibody – drug conjugates in breast cancer

**DOI:** 10.3389/fonc.2026.1755574

**Published:** 2026-05-29

**Authors:** Ajay Gogia

**Affiliations:** Medical Oncology, All India Institute of Medical Sciences, New Delhi, India

**Keywords:** antibody drug conjugate, breast cancer, management, prevention, toxicities

## Abstract

**Background:**

Antibody–drug conjugates (ADCs) have transformed breast-cancer therapeutics by combining targeted antibody delivery with potent cytotoxic payloads. With the expanding use of trastuzumab deruxtecan (T-DXd), sacituzumab govitecan (SG), and datopotamab deruxtecan (Dato-DXd) across HER2-positive, HER2-low, and hormone-receptor–positive disease, management of treatment-related toxicities has become a critical determinant of outcomes.

**Objective:**

To synthesize clinical-trial, post-marketing, and real-world data to create a unified, organ-system–based framework for anticipating, preventing, and managing toxicities associated with these ADCs.

**Methods:**

A targeted narrative review was conducted using PubMed, Embase, and ClinicalTrials.gov (January 2019 – December 2025). Phase II/III trials, meta-analyses, pharmacovigilance studies, and expert-consensus guidelines on ADC safety in breast cancer were included. Pre-clinical and non-breast ADC studies were excluded.

**Results:**

Comparative synthesis demonstrates overlapping but distinct toxicity signatures driven by payload, linker stability, and antigen expression. Integrating these findings, we propose practical algorithms for managing pulmonary, gastrointestinal, hematologic, ocular, and mucosal adverse events.

**Conclusion:**

This review consolidates evidence into a clinician-oriented reference for ADC toxicity management, emphasizing multidisciplinary coordination, early recognition, and system-specific mitigation strategies to enhance treatment safety and adherence.

## Introduction

The therapeutic landscape of breast cancer has witnessed immense developments over the last 2 decades since the inception of trastuzumab, a HER2-directed monoclonal antibody in clinical use (1). Dual HER2 blockade combining pertuzumab and trastuzumab has helped achieve a median overall survival of close to 5 years (2). CDK 4/6 inhibitors in early and metastatic settings have transformed treatment outcomes in endocrine-sensitive disease (3). Triple negative breast cancer, synonymous with lack of druggable targets, can now be managed with immune checkpoint inhibitors and PARP inhibitors (4). Antibody drug conjugates, or ADCs, are a promising cancer treatment approach that uses antibody targeting to deliver chemotherapy only to cancer cells. ADCs are increasingly employed in the treatment of various breast cancer subtypes, encompassing HR-positive, HER-2-positive, low, ultra-low, and TNBC. The US FDA has approved the 3 ADCs in the management of breast cancer named trastuzumab deruxtecan (T-DXd), sacituzumab govitecan-hziy (SG), and datopotamab deruxtecan (Dato-DXD) (**Table 1**) (5–13). While pivotal trials such as DESTINY-Breast 03/04/05/06/09/011, ASCENT, TROPiCS-02, and TROPION-Breast 01 have established remarkable efficacy, real-world implementation has revealed complex toxicity patterns that differ from those of conventional chemotherapy or immunotherapy. More recently, phase III ASCENT-03 and ASCENT-04 have moved SG into the first-line metastatic triple-negative breast cancer setting, underscoring the need for precise toxicity surveillance and supportive-care algorithms as antibody–drug conjugates are introduced earlier in the disease course. Current information on adverse-event management remains dispersed across package inserts and individual study reports, offering limited practical guidance for oncologists confronting multifaceted toxicities in routine care. Despite the growing clinical utility of antibody–drug conjugates (ADCs) in breast cancer, current evidence regarding toxicity management remains fragmented across individual clinical trials, prescribing information, and heterogeneous real-world reports. There is a lack of a unified, organ-system–based framework that integrates toxicity incidence, mechanisms, and practical management strategies across different ADCs. Furthermore, differences in toxicity profiles driven by payload, linker stability, and antigen expression are not systematically compared in existing literature, limiting their applicability in routine clinical decision-making.

**Table 1:** US FDA approvals of trastuzumab deruxtecan, sacituzumab govitecan and datopotamab deruxtecan.

US-FDA indication	Clinical trial	Date of indication approval
Trastuzumab deruxtecan		
HER2-positive (IHC 3+ or ISH positive) ABC after progression on anti-HER2-based regimen: in the metastatic setting, or in the (neo)adjuvant setting developing recurrence during or within six months of completing therapy.	Destiny – breast 03 (5)	Dec 20, 2019 (Accelerated); May 2022 (full approval)
HER2-low (IHC 1+ or IHC 2+/ISH-) advanced breast cancer after progression on chemotherapy: in the metastatic setting, or disease recurrence during or within 6 months of completing adjuvant chemotherapy.	Destiny – breast 04 (6)	Aug 5 2022
HR – positive, HER2-low (IHC 1+ or IHC 2+/ISH-) or HER2- ultralow (IHC 0 with membrane staining) advanced breast cancer, after progressing on: one or more endocrine therapies in the metastatic setting.	Destiny – breast 06 (7)	January 27 2025
HR+ve, advanced or metastatic breast cancer. Patients had received no previous chemotherapy or HER2-directed therapy for metastatic disease. One previous line of endocrine monotherapy was permitted in the context of metastatic disease.	Destiny – breast 09 (8)	Dec 15, 2025
High risk, HER2+ve, early breast cancer previously untreated, locally advanced or inflammatory breast cancer with a primary clinical tumor stage of ≥cT3 and cN0-3, or a primary tumor of any size with positive lymph node involvement (any cT and cN1-3). HER2-positive status [immunohistochemistry (IHC) 3+or *in situ* hybridization (ISH)+].	Destiny – breast 011 (9)	Still pending
HER2-positive breast cancer with residual invasive disease and node-positive disease at surgery or inoperable disease at diagnosis	Destiny – breast 05 (10)	Granted Breakthrough Therapy Designation (BTD) based ESMO 2025 symposium
Sacituzumab govitecan		
Advanced triple-negative breast cancer (mTNBC) who have received two or more prior systemic therapies, at least one of them for metastatic disease	ASCENT (11)	Apr 22 2020
HR – positive, HER2 – negative (IHC 0, IHC 1+ or IHC 2+/ISH–) advanced breast cancer post endocrine based therapy and at least two additional systemic therapies in the metastatic setting.	TROPiCS – 02 (12)	Feb 3 2023
Datopotamab deruxtecan		
HR – positive, HER2 – negative (IHC 0, IHC 1+ or IHC 2+/ISH-) advanced breast cancer who have received prior endocrine-based therapy and chemotherapy for unresectable or metastatic disease.	TROPION – Breast 01 (13)	January 17 2025

Approval status and indications compiled from corresponding pivotal trial publications and regulatory/prescribing documentation.

This review addresses this critical practice gap by integrating available clinical and real-world evidence across currently approved ADCs in breast cancer. Specifically, it aims to provide comparative insights into the incidence, severity, and mechanisms of common and serious toxicities; to organize toxicity management according to affected organ systems to enable side-by-side evaluation; to translate trial data and real-world experience into clinician-friendly approaches for early recognition, prevention, and intervention; and to identify current evidence gaps and future research priorities in ADC safety optimization.

## Methods

**Figure 1:**
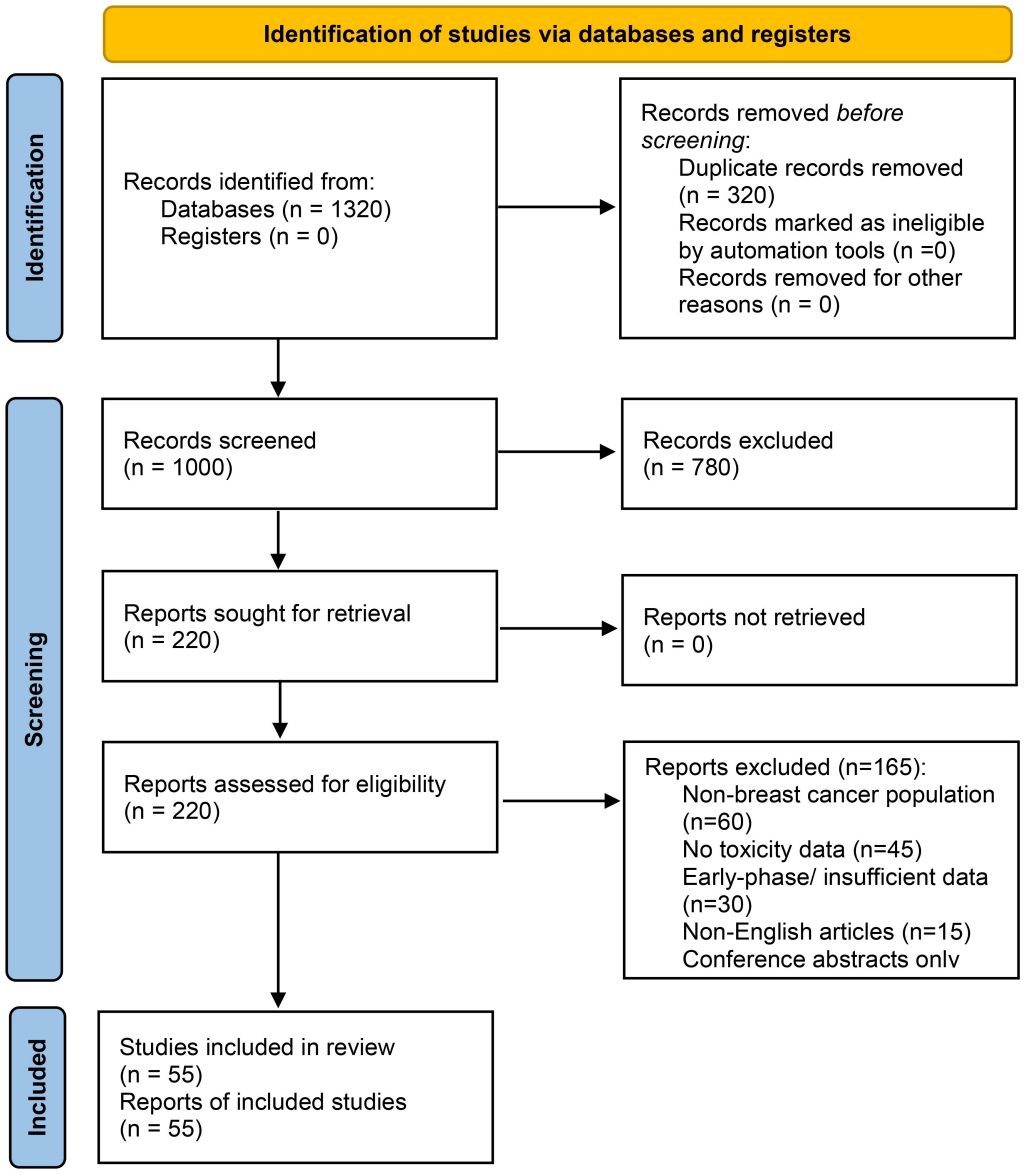
PRISMA 2020 flow diagram illustrating the process of study identification, screening, eligibility assessment, and inclusion.

This narrative review was conducted to consolidate evidence on the incidence, mechanisms, and management of toxicities associated with currently approved antibody–drug conjugates (ADCs) for breast cancer. A targeted literature search was performed in PubMed, Embase, and ClinicalTrials.gov from January 2019 through December 2025, using combinations of the keywords *“antibody–drug conjugate,” “toxicity,” “safety,” “breast cancer,”* and the individual drug names (*trastuzumab deruxtecan*, *sacituzumab govitecan*, *datopotamab deruxtecan*). Eligible sources included phase II/III clinical trials, pooled analyses, meta-analyses, post-marketing pharmacovigilance reports, and expert-consensus or guideline publications. Preclinical studies, early-phase non-breast-cancer trials, conference abstracts without peer-reviewed data, and non-English articles were excluded. Data from included studies were synthesized qualitatively to compare the frequency, severity, and organ-system distribution of adverse events and to develop evidence-based management approaches. T-DM1 was excluded because its toxicity spectrum—microtubule-inhibitor–related hepatic and thrombocytopenic effects—has been extensively characterized over the past decade and differs mechanistically from the topoisomerase I-based payloads of the newer ADCs. Where appropriate, findings were cross-referenced with FDA prescribing information and updated through recent publications up to December 2025. A total of 1320 records were identified through database searching (PubMed n=612, Embase n=476, ClinicalTrials.gov n=160) and additional sources (n=72). After removal of 320 duplicates, 1,000 records were screened based on title and abstract, of which 780 were excluded. A total of 220 full-text articles were assessed for eligibility. Of these, 165 were excluded due to the following reasons: non-breast cancer population (n=60), absence of toxicity-related outcomes (n=45), early-phase or insufficient data (n=30), non-English publications (n=15), and conference abstracts without peer-reviewed full text (n=15). Finally, 55 studies were included in the qualitative synthesis (**Figure 1**).

## Mechanism of action of ADC

ADCs operate by a complex, multifactorial mechanism of action. A monoclonal antibody, a cleavable or non-cleavable linker, and a cytotoxic payload make up the three primary parts of the ADC (14). The monoclonal antibody, component of the ADC is designed to identify and attach to a specific antigen that is overexpressed on the surface of cancer cells. Upon binding of the ADC to the target antigen, the complete complex undergoes internalization into cancer cells via receptor-mediated endocytosis. The ADC is transported to endosomes within cancer cells and subsequently fuses with lysosomes. This process initiates the enzymatic degradation of the linker, resulting in the release of the cytotoxic payload from the monoclonal antibody, resulting in its effect within the cancer cells (15). The mechanism of action of ADCs is summarized in **Table 2**. All three components of ADCs—monoclonal antibody (mAb), linker, and payload—can contribute to their toxicity; however, the payload is primarily responsible for this effect. **Table 3** presents the characteristics of three novel ADCs, including their antibodies, linkers, cytotoxic payloads, and pharmacokinetics (16–18).

**Table 2:** Mechanism of action of antibody-drug-conjugates.

Step	Key function	Mechanism of action	Clinical significance
1	Target antigen recognition	Monoclonal antibody binds to a tumor-specific antigen on cancer cell surface (e.g., HER2, TROP-2, CD30)	Ensures selectivity and minimizes damage to normal cells
2	Antigen–antibody binding	High-affinity binding of ADC to antigen on tumor cell membrane	Determines efficacy and tumor targeting
3	Internalization	ADC–antigen complex is internalized into the cell via endocytosis	Required for intracellular drug delivery
4	Endosomal–lysosomal trafficking	ADC is transported to lysosomes	Provides environment for linker cleavage
5	Linker cleavage	Linker is cleaved by enzymes or low pH (cleavable) or antibody is degraded (non-cleavable)	Controls timing and location of drug release
6	Payload release	Cytotoxic drug (payload) is released inside the cancer cell	Enables high intracellular drug concentration
7	Cytotoxic action	Payload causes DNA damage or microtubule inhibition	Leads to apoptosis and tumor cell death
8	Bystander effect (select ADCs)	Payload diffuses to nearby tumor cells	Kills antigen-low or heterogeneous tumor cells

**Table 3:** Comparison of attributes of ADCs in clinical use. (DAR: drug antibody ratio).

	Attributes	Trastuzumab deruxtecan (16)	Sacituzumab govitecan (17)	Datopotamab deruxtecan (18)
Antibody	Target	HER2	TROP2	TROP2
	Antibody type	IgG1	IgG1	IgG1
	DAR	~8:1	~8:1	~4:1
Linker	Linker	Tetrapeptide-based	Hydrolysable	Tetrapeptide-based
	Cleavable linker	Yes	Yes	Yes
	Linker cleavage trigger	Lysosomal cathepsis	Low pH	Lysosomal cathepsis
Payload	Payload	Deruxtecan	SN – 38	Deruxtecan
	Payload class	Topoisomerase I inhibitor	Topoisomerase I inhibitor	Topoisomerase I inhibitor
	Membrane permeable	Yes	Yes	Yes
Pharmacokinetics	Endocytosis mechanism	Caveolae – endocytosis mechanism	Clathrin – mediated endocytosis	Clathrin – mediated endocytosis
	Half – life	~7 days	~15 hours	~5 days
	Excretion	Biliary	Biliary	Biliary

Drug-specific pharmacologic characteristics adapted from the key preclinical/early translational references for each ADC (16–18).

## Generations of antibody-drug conjugates

Antibody–drug conjugates (ADCs) have evolved through multiple generations, with progressive improvements in linker stability, payload potency, and therapeutic index. First-generation ADCs, such as gemtuzumab ozogamicin, were limited by unstable linkers and off-target toxicity. Second-generation ADCs, including trastuzumab emtansine (T-DM1), incorporated more stable linkers and improved pharmacokinetics but retained limitations related to payload delivery and restricted bystander effect. T-DM1 was excluded as its toxicity profile, which is primarily characterized by hepatotoxicity and thrombocytopenia has been extensively established over the past decade and differs mechanistically from the topoisomerase I–based payloads of newer-generation ADCs. Third-generation ADCs, represented by trastuzumab deruxtecan, sacituzumab govitecan, and datopotamab deruxtecan, are characterized by higher drug-to-antibody ratios, cleavable linkers, and membrane-permeable payloads that enable a significant bystander effect. These structural and pharmacologic advancements translate into enhanced antitumor activity but are also associated with distinct toxicity profiles, particularly related to systemic payload exposure. Given these mechanistic differences, this review focuses on third-generation ADCs currently approved in breast cancer, as they represent the contemporary therapeutic landscape and exhibit unique and clinically relevant toxicity patterns.

## Broad overview of toxicity patterns of toxicities of ADCs

Antibody–drug conjugates (ADCs) used in breast cancer demonstrate characteristic toxicity patterns driven by their shared topoisomerase I payloads, linker stability, and antigen distribution. Pooled analyses indicate that any-grade adverse events occur in more than 90% of patients, with grade ≥3 toxicities in approximately 45–50%, highlighting the importance of structured toxicity assessment before detailed organ-specific management^19–20.^

Across the three approved ADCs, gastrointestinal and hematologic toxicities are the most prevalent, while pulmonary, mucosal, and ocular toxicities exhibit agent-specific patterns (**Table 4**).

**Table 4:** Comparison of all grade, common terminology criteria for adverse events (CTCAEversion 5) toxicities of ADCs from landmark clinical trials. NR: not reported (side effects in percentage %).

ADC	Trastuzumab deruxtecan			Sacituzumab govitecan		Datopotamab deruxtecan
Trial	DESTINY – breast 03 (5)	DESTINY – breast 04 (6)	DESTINY – breast 06 (7)	TROPiCS – 02 (8)	ASCENT (9)	TROPI on breast 01 (10)
Indication	HER2+	HER2 – low	HER2 – low & ultralow	HR+; HER2 – Negative	TNBC	HR+; HER2-Negative
Gastrointestinal						
Nausea	77	73	65.9	55	57	51.1
Vomiting	52.9	34	27.2	19	29	19.7
Constipation	37.7	21.3	NR	18	17	18.1
Diarrhea	33.5	22.4	23.7	57	59	7.5
Abdominal pain	24.9	NR	NR	13	11	NR
Stomatitis	23.3	NR	NR	NR	NR	50
Blood and lymphatic system						
Neutropenia	45.5	33.2	37.6	70	63	10.8
Anemia	38.1	33.2	NR	34	34	11.1
Leukopenia	34.2	23.2	23.3	14	16	7.2
Thrombocytopenia	31.5	23.7	NR	21	5	NR
General						
Fatigue	53.3	47.7	46.8	37	45	23.6
Alopecia	40.1	37.7	45.4	46	46	36.4
Decreased appetite	29	28.6	23.5	15	20	13.9
Neuropathy	13	NR	NR	9	1	NR
Adverse event of special interest						
Interstitial lung disease	16.7	10.2	5.3	0	0	1.4
Keratitis	NR	NR	NR	NR	NR	14.4

Adverse-event frequencies extracted from landmark clinical trial publications listed in the table columns and corresponding primary reports (5–7, 11–13).

*Gastrointestinal toxicity:* Nausea and vomiting remain the most frequent treatment-related AEs, affecting 70–75% of patients receiving T-DXd and 55–60% with SG or Dato-DXd. These events are largely attributed to topoisomerase-I payload exposure and are dose-dependent. However, diarrhea shows a divergent pattern: it is common with SG (≈57–59%) due to the SN-38 payload, less frequent with T-DXd (≈25–33%), and minimal with Dato-DXd (<10%) owing to its stable linker and limited systemic payload release. These distinctions highlight how linker chemistry modulates off-target gastrointestinal toxicity (19–21).

*Hematologic toxicity:* Myelosuppression, particularly neutropenia and anemia, represents a major class effect. SG demonstrates the highest incidence (neutropenia ≈ 63–70%, anemia ≈ 34%) consistent with its irinotecan-derived payload and black-box warning. In contrast, T-DXd produces neutropenia in roughly 35–45%, and Dato-DXd in <15% (18, 22). Thrombocytopenia is most prominent with T-DXd, whereas leukopenia and lymphopenia are relatively balanced across agents. These data suggest a gradient of marrow toxicity corresponding to the payload’s bioavailability and systemic exposure.

*Other organ specific toxicities:* Interstitial lung disease (ILD)/pneumonitis is a hallmark of T-DXd, occurring in 10–20% of patients overall, including up to 3% fatal events. Dato-DXd demonstrates a lower but notable ILD incidence (~1–2%), while SG shows negligible pulmonary toxicity. These findings support a payload- and exposure-related mechanism rather than a target-specific one. Mucosal and ocular events are largely confined to Dato-DXd, with stomatitis in ~50% and keratitis or dry-eye symptoms in ~15–20%. These effects likely stem from TROP-2 expression in mucosal and corneal epithelia and the compound’s linker stability (20, 21).

*Treatment Discontinuation and Dose Modifications:* Treatment discontinuation due to AEs occurs in 9–12% of patients on T-DXd, 6–8% on SG, and <5% on Dato-DXd. The most common reasons are ILD/pneumonitis for T-DXd, neutropenia or diarrhea for SG, and stomatitis for Dato-DXd. Dose reductions are reported in up to 20–25% of patients across all agents, primarily during early treatment cycles, emphasizing the importance of anticipatory supportive care and individualized dosing (22).

Collectively, these data underscore that ADC-related toxicity profiles are influenced not only by payload class but also by linker design, target expression, and pharmacokinetic behavior. Understanding these inter-agent distinctions allows clinicians to tailor monitoring protocols and pre-emptive interventions according to each ADC’s toxicity signature before delving into organ-specific management strategies. These overarching toxicity patterns provide a framework for understanding ADC-related adverse events; detailed organ-system–based toxicity profiles and management strategies are discussed in the subsequent section.

## Organ-system–based toxicity and their management

A structured organ-system–based approach to toxicities with trastuzumab deruxtecan (T-DXd), sacituzumab govitecan (SG), and datopotamab deruxtecan (Dato-DXd) is being presented below (21, 23–26). The systemic toxicity and management of all 3 ADCs are described in **Tables 5**–**8**.

**Table 5:** Trastuzumab deruxtecan: clinical monitoring and management of ILD/pneumonitis.

Monitor patients for and promptly investigate the signs and symptoms of ILD:					
Cough	Dyspnea		Fever		New or worsening respiratory symptoms
Talk to your patients to raise awareness and help identify symptoms					
Advise patients to contact their health care provider immediately for any of the symptoms shown above. Inform patients of the risks of severe, life-threatening, or fatal ILD.					
STEP 1: Monitor					
Rule out ILD/pneumonitis if a patient develops radiographic changes potentially consistent with ILD/pneumonitis or develops an acute onset of new or worsening pulmonary or other related signs/symptoms, such as dyspnea, cough, or fever.					
STEP 2: Confirm					
High-resolution CT. Pulmonologist consultation (infectious disease consultation as clinically indicated). Blood culture and CBC. Other blood tests could be considered as needed. Consider bronchoscopy and bronchoalveolar lavage if clinically indicated and feasible. PFTs and pulse oximetry. Arterial blood gasses, if clinically indicated. One blood sample collection for PK analysis as soon as ILD/pneumonitis is suspected, if feasible.					
STEP 3: Manage					
Grade 1		Grade 2		Grade 3	
Monitor and closely follow-up in 2 to 7 days for onset of clinical symptoms and pulse oximetry >Consider follow-up imaging in 1–2 weeks (or as clinically indicated) >Consider starting systemic steroids (e.g., at least 0.5 mg/kg/day prednisone or equivalent) until improvement, followed by gradual taper over at least 4 weeks If worsening of diagnostic observations despite initiation of corticosteroids, then follow Grade 2 guidelines — If patient is asymptomatic, then patient should still be considered as Grade 1 even if steroid treatment is given		Promptly start and treat with systemic steroids (e.g., at least 1 mg/kg/day prednisone or equivalent) for at least 14 days, then followed by a gradual taper over at least 4 weeks Monitor symptoms closely Reimage as clinically indicated If worsening or no improvement in clinical or diagnostic observations in 5 days, —Consider increasing dose of steroids (e.g., 2 mg/kg/day prednisone or equivalent) and administration may be switched to intravenous (e.g., methylprednisolone) —Reconsider additional work-up for alternative etiologies as described above —Escalate care as clinically indicated		Hospitalization required Promptly initiate empiric high-dose methylprednisolone IV treatment (e.g., 500–1000 mg/day for 3 days), followed by at least 1.0 mg/kg/day of prednisone (or equivalent) for at least 14 days followed by a gradual taper over at least 4 weeks Reimage as clinically indicated If still no improvement within 3 to 5 days, —Reconsider additional work-up for alternative etiologies as described above —Consider other immuno-suppressants and/or treat per local practice	

Adapted from multidisciplinary guidance on trastuzumab deruxtecan-related ILD/pneumonitis and the current ENHERTU prescribing information (27–30).

**Table 6:** Clinical management of trastuzumab deruxtecan induced toxicities trastuzumab deruxtecan: dose reduction schedule.

Recommended starting dose		5.4 mg/kg	
First dose reduction		4.4 mg/kg	
Second dose reduction		3.2 mg.kg	
Requirement for further dose reduction		Discontinue treatment	
Trastuzumab deruxtecan: dose modification			
Adverse reaction	Severity		Treatment modification
Interstitial lung disease (ILD)/pneumonitis	Asymptomatic ILD/pneumonitis (Grade 1)		Interrupt until resolved to Grade 0, then: if resolved in 28 days or less from date of onset, maintain dose. if resolved in greater than 28 days from date of onset, reduce dose one level. consider corticosteroid treatment as soon as ILD/pneumonitis is suspected.
	Symptomatic ILD/pneumonitis (Grade 2 or greater)		Permanently discontinue. Promptly initiate corticosteroid treatment as soon as ILD/pneumonitis is suspected.
Neutropenia	Grade 3 (less than 1.0 to 0.5 x 10^9^/L)		Interrupt until resolved to Grade 2 or less, then maintain dose.
	Grade 4 (less than 0.5 x 10^9^/L)		Interrupt until resolved to Grade 2 or less. Reduce dose by one level.
Febrile neutropenia	Absolute neutrophil count of less than 1.0 x 10^9^/L and temperature greater than 38.3 °C or a sustained temperature of 38 °C or greater for more than one hour		Interrupt until resolved. Reduce dose by one level.
Thrombocytopenia	Grade 3 (platelets less than 50 to 25 x 10^9^/L)		Interrupt until resolved to Grade 1 or less, then maintain dose.
	Grade 4 (platelets less than 25 x 10^9^/L)		Interrupt until resolved to Grade 1 or less. Reduce dose by one level.
Left ventricular dysfunction	LVEF greater than 45% and absolute decrease from baseline is 10% to 20%		Continue treatment.
	LVEF 40% to 45%	And absolute decrease from baseline is less than 10%	Continue treatment. Repeat LVEF assessment within 3 weeks.
		And absolute decrease from baseline is 10% to 20%	Interrupt. Repeat LVEF assessment within 3 weeks. If LVEF has not recovered to within 10% from baseline, permanently discontinue. If LVEF recovers to within 10% from baseline, resume treatment at the same dose.
	LVEF less than 40% or absolute decrease from baseline is greater than 20%		Interrupt. Repeat LVEF assessment within 3 weeks. If LVEF of less than 40% or absolute decrease from baseline of greater than 20% is confirmed, permanently discontinue.
	Symptomatic congestive heart failure (CHF)		Permanently discontinue.

Dose reductions and dose-modification recommendations adapted from ENHERTU prescribing information (30).

**Table 7:** Sacituzumab govitecan: dosage reduction schedule and dose modifications.

Sacituzumab govitecan: dose reduction schedule		
Recommended starting dose	10 mg/kg once weekly on Days 1 and 8 of 21-day treatment cycles	
First dose reduction	Reduce to 7.5 mg/kg	
Second dose reduction	Reduce to 5 mg/kg	
Requirement for further dose reduction	Permanently discontinue	
Sacituzumab govitecan: dose modification		
Adverse reaction	Severity	Treatment modifications
Neutropenia	Grade 3–4 neutropenia (ANC <1000/mm^3^) or febrile neutropenia	Withhold until ANC ≥1500/mm^3^ for Day 1 dose or ANC ≥1000/mm^3^ for Day 8 Dose. Administer G-CSF during treatment as clinically indicated. Reduce one dose level for each occurrence of febrile neutropenia or prolonged Grade 3–4 neutropenia or discontinue according to dose reduction schedule.
Nausea/Vomiting/Diarrhea	Grade 3–4 nausea, vomiting or diarrhea that is not controlled with antiemetics or anti-diarrheal agents	Withhold until resolved to ≤ Grade 1. Reduce one dose level with each occurrence, or. discontinue according to dose reduction schedule.
Infusion-Related Reaction	Grade 1–3 infusion-related reactions	Slow infusion rate or interrupt the infusion.
	Grade 4 infusion-related reactions	Discontinue.

Dose reductions and dose-modification recommendations adapted from TRODELVY prescribing information; recommendations regarding primary G-CSF prophylaxis are additionally supported by PRIMED (31, 32).

**Table 8:** Datopotamab deruxtecan: dosage reduction schedule and dose modifications.

Datopotamab deruxtecan: dose reduction schedule			
Recommended starting dose		6 mg/kg	
First dose reduction		4 mg/kg (up to a maximum of 360 mg for patients ≥90 kg)	
Second dose reduction		3 mg/kg (up to a maximum of 270 mg for patients ≥90 kg)	
Requirement for further dose reduction		Permanently discontinue	
Datopotamab deruxtecan: dose modification			
Adverse reaction	Severity		Treatment modifications
Interstitial Lung Disease (ILD)/Pneumonitis	Asymptomatic ILD/pneumonitis Grade 1		Withhold until ILD/pneumonitis is completely resolved, then: if resolved in ≤28 days, maintain current dose. if resolved in >28 days, reduce one dose level. >Consider corticosteroids as soon as ILD/pneumonitis is suspected.
	Symptomatic ILD/pneumonitis Grade 2 or greater		Permanently discontinue. Administer corticosteroids as soon as ILD/pneumonitis is suspected.
Keratitis	Nonconfluent superficial keratitis		Monitor
	Confluent superficial keratitis, a cornea epithelial defect, or 3-line or more loss in best corrected visual acuity		Withhold until improved or resolved, then maintain at same dose level or consider dose reduction.
	Corneal ulcer or stromal opacity or best corrected distance visual acuity 20/200 or worse		Withhold until improved or resolved, then reduce by one dose level.
	Corneal perforation		Permanently discontinue.
Stomatitis	Grade 1		Optimize prophylactic and supportive medications.
	Grade 2		Withhold until resolved to < Grade 1. Restart at the same dose level for first occurrence. Consider restarting at reduced dose level if recurrent.
	Grade 3		Withhold until resolved to ≤Grade 1. Restart at reduced dose level.
	Grade 4		Permanently discontinue.
Infusion-Related Reactions (IRR)	Grade 1		Reduce infusion rate by 50% if IRR is suspected and monitor patient closely.
	Grade 2		Interrupt infusion and administer supportive care medications. If the event resolves or improves to Grade 1, restart the infusion at 50% rate. Administer all subsequent infusions at the reduced rate.
	Grade 3 or 4		Permanently discontinue.

Dose reductions and dose-modification recommendations adapted from DATROWAY prescribing information; ocular toxicity reporting may additionally be standardized using the proposed interspecialty ocular adverse-event grading scales (33, 34).

### Pulmonary toxicity

Pulmonary toxicities, particularly interstitial lung disease (ILD) and pneumonitis, are hallmark adverse events with T-DXd, occurring in approximately 10–20% of patients, with a fatality rate near 3% (26, 35–37). These events are considerably less frequent with Dato-DXd (1–2%) and exceedingly rare with SG (38). The mechanism involves target-independent uptake of the deruxtecan payload by alveolar macrophages, producing diffuse alveolar damage; risk is higher in patients with pre-existing lung disease and those of East-Asian ancestry (39). Additional risk factors that warrant heightened vigilance include baseline oxygen saturation <95%, moderate or severe renal impairment, and pre-existing lung comorbidity; pooled analyses and multidisciplinary guidance have also identified a higher observed incidence in Japanese and East-Asian populations. Preventive strategies include baseline high-resolution CT imaging, pulse oximetry, and clinical assessment before each cycle. In routine practice, a baseline HRCT should be obtained before treatment initiation, with surveillance chest CT performed approximately every 6–12 weeks during the first 6–12 months of therapy; pulse oximetry and directed symptom review should be documented at every visit, and more frequent imaging may be considered in higher-risk patients (40). Patients should be counselled to report early respiratory symptoms such as cough, dyspnea, or fever. When ILD or pneumonitis is suspected, ADC therapy must be interrupted and corticosteroids promptly started. Trastuzumab deruxtecan must be permanently discontinued in all patients with Grade ≥2 ILD/pneumonitis (41). Grade 1 events are managed with oral prednisone 0.5 mg/kg daily for ≥14 days followed by a taper; Grade 2 events require 1 mg/kg daily until resolution. For Grade ≥3 events, IV methylprednisolone 500–1000 mg daily × 3 days followed by tapering oral therapy is indicated, and permanent discontinuation of the ADC is advised (42). Re-challenge is considered only after full resolution of Grade 1 toxicity within four weeks (25, 43).

### Gastrointestinal toxicity

Gastrointestinal toxicities are the most common treatment-emergent events across ADCs. Nausea and vomiting occur frequently—T-DXd (70–75%), SG (55–60%), Dato-DXd (~50%)—and are usually delayed in onset owing to systemic payload exposure.^37-39.^ Standard prophylaxis includes a 5-HT_3_ receptor antagonist with dexamethasone, adding an NK_1_ receptor antagonist for high-risk patients; olanzapine 5 mg nightly for four days can be used for refractory cases^.40^ Diarrhea is a distinguishing feature of SG, affecting about 59% of patients (10% Grade ≥3) due to SN-38–induced mucosal injury and oxidative stress (44). Management includes ruling out infection, initiating loperamide (4 mg initially, then 2 mg after each loose stool; max 16 mg/day), and escalating to octreotide (100–150 µg SC three times daily) with hydration if persistent beyond 24 hours (45). Acute cholinergic diarrhea may respond to atropine 0.4 mg IV every 15 min up to two doses (46). In the PRIMED study, primary prophylaxis with G-CSF plus loperamide reduced the incidence and severity of SG-related neutropenia and diarrhea, supporting upfront prophylaxis in selected patients (18). Stomatitis and oral mucositis, common with Dato-DXd (~50%), arise from on-target TROP-2 expression in the oral mucosa (47). Prophylaxis with betamethasone mouthwash (0.1 mg/mL × 4 daily from day 1) and oral cryotherapy during infusion is recommended.^46.^ Treatment should be withheld for Grade ≥2 mucositis until recovery to ≤ Grade 1; resume at reduced dose upon recurrence and discontinue for Grade 4 events (48).

### Hematologic toxicity

Hematologic toxicities occur with all ADCs but are most pronounced with SG (neutropenia 63–70%), intermediate with T-DXd (~40%), and minimal with Dato-DXd (<15%) (23). These arise from SN-38–mediated marrow suppression and topoisomerase I inhibition (49). Complete blood counts should precede each cycle. For Grade 3–4 neutropenia, hold treatment until ANC ≥1500/mm³ (Day 1) or ≥1000/mm³ (Day 8), reducing one level for recurrence (50). Febrile neutropenia requires interruption, empiric antibiotics, and G-CSF; anemia and thrombocytopenia are managed with transfusion and dose delays (51). Primary or secondary G-CSF prophylaxis is recommended for recurrent Grade ≥3 neutropenia. Primary prophylaxis with G-CSF should also be considered from cycle 1 in patients at increased risk of febrile neutropenia, including older patients, those with previous neutropenia, poor performance status, organ dysfunction, or multiple comorbidities (51).

### Mucocutaneous and ocular toxicity

Mucocutaneous and ocular toxicities are characteristic of Dato-DXd and result from TROP-2 expression in epithelial tissues combined with high linker stability (52). Ocular events—dry eye (22%), keratitis (14%), tearing (6%)—often present early. Because conventional CTCAE ocular terms may mix signs and symptoms, recently proposed consensus grading scales separate visual acuity, eye symptoms, cornea, conjunctiva/sclera, anterior chamber, and retina/posterior segment findings, each with associated dose-modification recommendations; this framework may improve consistency in reporting and management of Dato-DXd-related ocular toxicity. Accordingly, ophthalmologic assessment should document objective corneal findings separately from subjective symptoms rather than relying on umbrella terms alone (53). Prevention includes liberal use of artificial tears (≥4 times daily), avoidance of contact lenses, and nightly lubricating ointment (47). For Grade ≥2 keratitis, withhold until recovery, reduce one dose level if recurrent, and discontinue permanently for corneal ulceration or perforation (47). Alopecia, affecting ~40–45% of patients across ADCs, should be discussed pre-treatment, with optional scalp-cooling measures (54).

### Cardiac toxicity

Cardiac toxicity is unique to T-DXd owing to its HER2-targeted antibody component and is infrequent (~1.5–2% LVEF decline; rare heart failure). This mirrors trastuzumab-associated cardiomyopathy. Baseline and three-monthly echocardiograms are recommended; interrupt treatment if LVEF < 45% or decreases > 10% from baseline and discontinue permanently for symptomatic heart failure.

### Reproductive and embryo-fetal toxicity

Reproductive and embryo-fetal toxicity are recognized risks with T-DXd and SG, both carrying black-box warnings. Their payloads may cause DNA damage during organogenesis. Women should maintain effective contraception during therapy and for seven months after T-DXd or six months after SG; men should continue contraception during treatment and for four months after T-DXd or three months after SG. Pregnancy and lactation are contraindicated.

A comprehensive, integrated strategy for toxicity prevention and management is vital to ensure treatment continuity and optimize outcomes. Primary and secondary prevention of novel ADCs are described in **Table 9**. Proactive patient and staff education on early symptom recognition—cough, dyspnea, diarrhea, mucositis, and visual changes—enables timely intervention before complications escalate. Baseline evaluations should include HRCT, echocardiography, complete blood counts, hepatic and renal function tests, and ophthalmic examination for patients receiving Dato-DXd. Close multidisciplinary collaboration among pulmonology, dermatology, ophthalmology, nutrition, and supportive-care teams enhances quality of care. Guiding principles include withholding therapy for Grade ≥2 non-hematologic or Grade 3 hematologic events, implementing 25–50% dose reductions for recurrent toxicities, and discontinuing treatment permanently for life-threatening or unresolving adverse effects. Incorporating structured documentation and patient-reported-outcome monitoring into routine follow-up allows early signal detection, promotes adherence, and reinforces the overall safety and tolerability of ADC therapy in breast-cancer management.

**Table 9:** Primary and secondary prophylaxis of novel antibody drug conjugates.

Toxicity	Common ADCs involved	Primary prophylaxis (before toxicity)	Secondary prophylaxis (after toxicity)
Neutropenia/myelosuppression	Sacituzumab govitecan, T-DXd	Baseline CBC; avoid overlapping myelotoxic drugs; patient education on fever	G-CSF (secondary); dose delay/reduction; treatment interruption
Diarrhea	Sacituzumab govitecan	Early counseling; prescribe antidiarrheals in advance; hydration advice	Aggressive loperamide; IV fluids; dose modification
Nausea/vomiting	Sacituzumab govitecan, T-DXd, Dato DXd	Prophylactic antiemetics (moderate emetogenic risk) 5-HT3 antagonist,NK1antagonist, steroids	Escalation to combination antiemetic regimens
Interstitial lung disease (ILD)/pneumonitis	Trastuzumab deruxtecan	Baseline CT chest; pulmonary history; patient education; scheduled imaging	Immediate interruption; systemic corticosteroids; permanent discontinuation if ≥ grade 2
Peripheral neuropathy	T-DM1, MMAE-based ADCs	Baseline neurologic assessment; avoid neurotoxic drugs	Dose delay/reduction; discontinuation if persistent or severe
Infusion-related reactions	All ADCs (uncommon)	Antihistamines ± antipyretics (selected patients)	Slower infusion rate; enhanced premedication
Fatigue/asthenia	All ADCs	Counseling; activity planning	Dose adjustment; supportive care
Ocular toxicity (rare, ADC-specific)	Dato-DXd	Lubricating eye drops; baseline eye exam (if indicated)	Ophthalmology referral; treatment modification
Stomatitis	Dato-DXd	Steroid mouth wash	Ice chips

Primary and secondary prophylaxis recommendations synthesized from ENHERTU, TRODELVY, and DATROWAY prescribing information and the PRIMED study (30–32, 34).

## Discussion

The increasing integration of antibody–drug conjugates into breast-cancer therapy has redefined treatment sequencing and toxicity-management paradigms. Although T-DXd, SG, and Dato-DXd share topoisomerase I payloads, their distinct linkers, target antigens, and pharmacokinetics create clinically meaningful differences in safety and tolerability. Understanding these differences enables individualized drug selection and rational sequencing to optimize benefit while minimizing harm.

Treatment-selection considerations are primarily guided by tumor biology and prior therapy but should also account for patient-specific toxicity risks. T-DXd remains preferred for HER2-positive and HER2-low disease owing to superior efficacy demonstrated in DESTINY-Breast 03 and 04; however, its risk of interstitial lung disease warrants caution in patients with underlying pulmonary dysfunction or prior thoracic irradiation. In contrast, SG offers a valuable option for triple-negative and hormone-receptor–positive/HER2-negative disease but requires vigilance for neutropenia and diarrhea, particularly in patients with limited marrow reserve (55–57). Dato-DXd, characterized by lower hematologic and pulmonary toxicity but higher mucosal and ocular adverse events, may be advantageous for patients with prior myelosuppression or respiratory compromise. Selecting among these agents thus entails balancing oncologic efficacy with each patient’s comorbidity profile, prior treatment toxicities, and preferences.

Sequencing strategies are an emerging challenge as ADCs move earlier in treatment algorithms. Although prospective data are limited, cross-resistance appears incomplete, suggesting that sequential use of ADCs with differing targets or linkers may retain efficacy. When sequencing, clinicians should consider the potential for overlapping toxicities: for example, transitioning from T-DXd to Dato-DXd requires careful pulmonary monitoring to detect residual subclinical ILD, whereas switching from SG to Dato-DXd necessitates recovery from neutropenia and mucositis before initiation. Real-world evidence and recently reported phase III trials, including ASCENT-03 and ASCENT-04/KEYNOTE-D19 for triple-negative breast cancer, are expected to clarify optimal sequencing frameworks and identify predictors of cross-toxicity and cumulative burden.

Management in special populations, particularly older adults (≥ 65 years), demands an individualized approach integrating geriatric assessment, comorbidity screening, and functional status evaluation. Age-related declines in hepatic and renal clearance can prolong payload exposure, increasing risk of cytopenias, diarrhea, and fatigue. Dose intensity may need adjustment, but empirical dose reductions should be balanced against the potential loss of efficacy. Early nutritional support, mobility preservation, and polypharmacy review are integral to minimizing treatment-related morbidity in this group.

Current knowledge derives largely from pivotal clinical trials with limited long-term follow-up and under-representation of elderly and comorbid populations. There is a need for validated predictive biomarkers for toxicity susceptibility, mechanistic studies to elucidate off-target effects, and harmonized grading systems that better capture cumulative and chronic ADC toxicities. Real-world data on rechallenge, dose re-escalation, and multidisciplinary management models will further refine practice. Integrating systematic patient-reported outcome monitoring and longitudinal registries will be key to advancing evidence-based toxicity mitigation and informing next-generation ADC design with improved therapeutic indices.

## Conclusion

Antibody–drug conjugates (ADCs) have redefined breast-cancer therapeutics by combining targeted precision with potent cytotoxic activity. However, their success depends as much on toxicity management as on tumor control. Hematologic and gastrointestinal adverse events remain among the most frequent and clinically significant complications, but these can often be mitigated through dose modifications, treatment delays, and timely supportive care. Equally important is the early detection of serious toxicities such as interstitial lung disease or pneumonitis, where prompt recognition and intervention can substantially improve outcomes. Systematic screening, close vigilance, and education of both patients and the multidisciplinary oncology team are central to safe delivery. Collaboration with subspecialists—including pulmonologists, dermatologists, and ophthalmologists—further strengthens the management of organ-specific toxicities and continuity of care.

Despite meaningful advances, key evidence gaps persist. Predictive biomarkers for toxicity susceptibility, harmonized grading systems for ADC-related adverse events, and robust data on sequencing strategies are urgently needed. Integrating patient-reported outcomes, mechanistic studies, and real-world registries will be critical to refining evidence-based frameworks for toxicity mitigation and individualized therapy optimization. Looking forward, innovation is extending beyond cytotoxic payloads toward next-generation constructs such as immunostimulatory antibody conjugates (ISACs) that harness innate immune activation through toll-like receptor 7/8 or stimulator-of-interferon-genes agonists, as well as ADCs targeting elements of the tumor microenvironment, including T lymphocytes and fibroblasts (57–63). These advances may generate distinct toxicity signatures, underscoring the continued need for multidisciplinary research and careful clinical translation. Collectively, proactive management, cross-specialty collaboration, and ongoing innovation will determine the extent to which ADCs fulfill their promise of maximizing efficacy while minimizing harm in breast-cancer care.
